# MRI Radiomic Signature of White Matter Hyperintensities Is Associated With Clinical Phenotypes

**DOI:** 10.3389/fnins.2021.691244

**Published:** 2021-07-12

**Authors:** Martin Bretzner, Anna K. Bonkhoff, Markus D. Schirmer, Sungmin Hong, Adrian V. Dalca, Kathleen L. Donahue, Anne-Katrin Giese, Mark R. Etherton, Pamela M. Rist, Marco Nardin, Razvan Marinescu, Clinton Wang, Robert W. Regenhardt, Xavier Leclerc, Renaud Lopes, Oscar R. Benavente, John W. Cole, Amanda Donatti, Christoph J. Griessenauer, Laura Heitsch, Lukas Holmegaard, Katarina Jood, Jordi Jimenez-Conde, Steven J. Kittner, Robin Lemmens, Christopher R. Levi, Patrick F. McArdle, Caitrin W. McDonough, James F. Meschia, Chia-Ling Phuah, Arndt Rolfs, Stefan Ropele, Jonathan Rosand, Jaume Roquer, Tatjana Rundek, Ralph L. Sacco, Reinhold Schmidt, Pankaj Sharma, Agnieszka Slowik, Alessandro Sousa, Tara M. Stanne, Daniel Strbian, Turgut Tatlisumak, Vincent Thijs, Achala Vagal, Johan Wasselius, Daniel Woo, Ona Wu, Ramin Zand, Bradford B. Worrall, Jane M. Maguire, Arne Lindgren, Christina Jern, Polina Golland, Grégory Kuchcinski, Natalia S. Rost

**Affiliations:** ^1^J. Philip Kistler Stroke Research Center, Massachusetts General Hospital, Boston, MA, United States; ^2^Inserm, CHU Lille, U1172 - LilNCog (JPARC) - Lille Neurosciences and Cognition, University of Lille, Lille, France; ^3^A. A. Martinos Center for Biomedical Imaging, Massachusetts General Hospital, Harvard Medical School, Boston, MA, United States; ^4^Computer Science and Artificial Intelligence Laboratory, Massachusetts Institute of Technology, Cambridge, MA, United States; ^5^Division of Preventive Medicine, Department of Medicine, Brigham and Women’s Hospital and Harvard Medical School, Boston, MA, United States; ^6^CNRS, Institut Pasteur de Lille, US 41 - UMS 2014 - PLBS, Lille, France; ^7^Department of Medicine, Division of Neurology, University of British Columbia, Vancouver, BC, Canada; ^8^Department of Neurology, University of Maryland School of Medicine and Veterans Affairs Maryland Health Care System, Baltimore, MD, United States; ^9^School of Medical Sciences, University of Campinas (UNICAMP) and the Brazilian Institute of Neuroscience and Neurotechnology (BRAINN), Campinas, Brazil; ^10^Department of Neurosurgery, Geisinger, Danville, PA, United States; ^11^Research Institute of Neurointervention, Paracelsus Medical University, Salzburg, Austria; ^12^Division of Emergency Medicine, Washington University School of Medicine, St. Louis, MO, United States; ^13^Department of Neurology, Washington University School of Medicine and Barnes-Jewish Hospital, St. Louis, MO, United States; ^14^Institute of Biomedicine, Sahlgrenska Academy at University of Gothenburg, Gothenburg, Sweden; ^15^Department of Neurology, Neurovascular Research Group (NEUVAS), Institut Hospital del Mar d’Investigacions Mèdiques (IMIM), Universitat Autonoma de Barcelona, Barcelona, Spain; ^16^Department of Neurosciences, Experimental Neurology and Leuven Research Institute for Neuroscience and Disease (LIND), KU Leuven – University of Leuven, Leuven, Belgium; ^17^VIB, Vesalius Research Center, Laboratory of Neurobiology, Department of Neurology, University Hospitals Leuven, Leuven, Belgium; ^18^School of Medicine and Public Health, University of Newcastle, Newcastle, NSW, Australia; ^19^Department of Neurology, John Hunter Hospital, Newcastle, NSW, Australia; ^20^Division of Endocrinology, Diabetes and Nutrition, Department of Medicine, University of Maryland School of Medicine, Baltimore, MD, United States; ^21^Department of Pharmacotherapy and Translational Research and Center for Pharmacogenomics, University of Florida, Gainesville, FL, United States; ^22^Department of Neurology, Mayo Clinic, Jacksonville, FL, United States; ^23^Centogene AG, Rostock, Germany; ^24^Department of Neurology, Clinical Division of Neurogeriatrics, Medical University of Graz, Graz, Austria; ^25^Henry and Allison McCance Center for Brain Health, Center for Genomic Medicine, Massachusetts General Hospital, Boston, MA, United States; ^26^Department of Neurology and Evelyn F. McKnight Brain Institute, Miller School of Medicine, University of Miami, Miami, FL, United States; ^27^Institute of Cardiovascular Research, Royal Holloway University of London (ICR2UL), Egham, United Kingdom; ^28^Ashford and St. Peter’s Hospitals, Chertsey and Ashford, United Kingdom; ^29^Department of Neurology, Jagiellonian University Medical College, Krakow, Poland; ^30^Division of Neurocritical Care and Emergency Neurology, Department of Neurology, Helsinki University Central Hospital, Helsinki, Finland; ^31^Department of Clinica Neuroscience, Institute of Neuroscience and Physiology, Sahlgrenska Academy at University of Gothenburg, Gothenburg, Sweden; ^32^Department of Neurology, Sahlgrenska University Hospital, Gothenburg, Sweden; ^33^Stroke Division, Florey Institute of Neuroscience and Mental Health, Department of Neurology Austin Health, Heidelberg, VIC, Australia; ^34^Department of Radiology, University of Cincinnati College of Medicine, Cincinnati, OH, United States; ^35^Department of Clinical Sciences Lund, Radiology, Lund University, Lund, Sweden; ^36^Department of Radiology, Neuroradiology, Skåne University Hospital, Malmö, Sweden; ^37^Department of Neurology and Rehabilitation Medicine, University of Cincinnati College of Medicine, Cincinnati, OH, United States; ^38^Department of Neurology, Geisinger, Danville, PA, United States; ^39^Department of Neurology and Public Health Sciences, University of Virginia, Charlottesville, VA, United States; ^40^Faculty of Health, University of Technology Sydney, Ultimo, NSW, Australia; ^41^Department of Neurology and Rehabilitation Medicine, Skåne University Hospital, Lund, Sweden; ^42^Department of Clinical Sciences Lund, Neurology, Lund University, Lund, Sweden

**Keywords:** stroke, cerebrovascular disease (CVD), MRI, radiomics, machine learning, brain health

## Abstract

**Objective:**

Neuroimaging measurements of brain structural integrity are thought to be surrogates for brain health, but precise assessments require dedicated advanced image acquisitions. By means of quantitatively describing conventional images, radiomic analyses hold potential for evaluating brain health. We sought to: (1) evaluate radiomics to assess brain structural integrity by predicting white matter hyperintensities burdens (WMH) and (2) uncover associations between predictive radiomic features and clinical phenotypes.

**Methods:**

We analyzed a multi-site cohort of 4,163 acute ischemic strokes (AIS) patients with T2-FLAIR MR images with total brain and WMH segmentations. Radiomic features were extracted from normal-appearing brain tissue (brain mask–WMH mask). Radiomics-based prediction of personalized WMH burden was done using ElasticNet linear regression. We built a radiomic signature of WMH with stable selected features predictive of WMH burden and then related this signature to clinical variables using canonical correlation analysis (CCA).

**Results:**

Radiomic features were predictive of WMH burden (*R*^2^ = 0.855 ± 0.011). Seven pairs of canonical variates (CV) significantly correlated the radiomics signature of WMH and clinical traits with respective canonical correlations of 0.81, 0.65, 0.42, 0.24, 0.20, 0.15, and 0.15 (FDR-corrected *p*-values_*CV*__1__–__6_ < 0.001, *p*-value_*CV*__7_ = 0.012). The clinical CV1 was mainly influenced by age, CV2 by sex, CV3 by history of smoking and diabetes, CV4 by hypertension, CV5 by atrial fibrillation (AF) and diabetes, CV6 by coronary artery disease (CAD), and CV7 by CAD and diabetes.

**Conclusion:**

Radiomics extracted from T2-FLAIR images of AIS patients capture microstructural damage of the cerebral parenchyma and correlate with clinical phenotypes, suggesting different radiographical textural abnormalities per cardiovascular risk profile. Further research could evaluate radiomics to predict the progression of WMH and for the follow-up of stroke patients’ brain health.

## Introduction

White matter hyperintensities (WMH) are a cardinal manifestation of small vessel disease (SVD) (Wardlaw et al., 2013). Increased WMH burden is associated with incident ischemic stroke and worse clinical outcome (Arsava et al., 2009). Beyond ischemic stroke, WMH are also associated with vascular cognitive impairment and dementia (Au et al., 2006). WMH prevalence increases with age but is also directly influenced by individual small vessel risk factors: the aggregation of cardiovascular risk factors leads to an increased WMH burden (Wardlaw et al., 2015). Hence, WMH are an imaging biomarker of brain health suggestive of neurodegeneration beyond normal brain aging (Brugulat-Serrat et al., 2019).

Structural injury of the brain has been shown to occur at the macrostructural level, in the form of WMH, but also at the microstructural level. Advanced diffusion tensor imaging (DTI) studies have shown an age-related loss of parenchymal microstructural integrity in normal-appearing white matter (NAWM) (Etherton et al., 2017a). Furthermore, perfusion-weighted imaging (PWI)-based research has also revealed age-related alterations of the blood-brain barrier with increased contrast agents’ leakage (Topakian et al., 2010). However, such microstructural injuries are not visualized with conventional structural MRI sequences, and as DTI and PWI require special acquisition times, the outlined imaging biomarkers are not currently used in clinical routine for SVD patients. Consequently, we are in need of conventional MRI-based methodologies that better quantify SVD and brain health to ensure a widespread application and translation to clinical practice.

Radiomic analyses cover a broad ensemble of high-throughput quantification methods applicable to digitalized medical images (Gillies et al., 2015). These methods automatically extract high-dimensional data, called radiomic features, by describing a given region of interest by its size, shape, histogram, and relationship between voxels. Because these techniques can capture slight differences in intensities and patterns that would remain undetected to a human reader, radiomics bear the potential to describe neuroimaging beyond what meets the naked eye, and thus might help to phenotype SVD (Lambin et al., 2012). Conceivably, they may identify early underlying brain injury at the individual level with rapid clinical translatability and thus enhance personalized care in stroke and SVD.

The aim of the current study was to assess the structural integrity of the brain using a texture analysis approach and to understand the infra-radiological footprint of WMH by exploring its relationship with cardiovascular risk profiles. To do so, we analyzed 4,163 T2 FLAIR images from a large multi-site international collaborative effort studying stroke and WMH. We sought to (a) build a robust radiomic signature of the subvisible manifestations of WMH and (b) to apply canonical correlation analysis (CCA) to investigate the relation between this latent textural expression in relation to sociodemographic information and cardiovascular risk factors, providing a potentially novel approach to improve SVD and stroke care.

## Materials and Methods

### Participants

We reviewed all ischemic stroke patients included in the MRI-GENetics Interface Exploration (MRI-GENIE) study, a large international multi-site collaboration of 20 sites gathering clinical, MRI imaging, and genetic data, built on top of the NINDS Stroke Genetics Network (SiGN) study. Both study design, data collection protocols, and populations have been previously described (Giese et al., 2020).

### Ethics

The MRI-GENIE project has been approved by the MGH Institutional Review Board (IRB, Protocol #: 2001P001186 and Protocol #: 2003P000836), as well as ethics boards of the collaborating institutions. All participants or health care proxy provided signed informed consent.

### Data Collection and Neuroimaging Pre-processing

Clinical data were acquired within the SiGN study and comprised information on age, sex, hypertension (HTN), history of smoking, diabetes mellitus (DM), atrial fibrillation (AF). Among the 6,627 patients included across 20 sites, FLAIR images were available for 6,389 patients. Axial T2-FLAIR images were acquired between 2003 and 2011 within 48 h of the initial stroke. They had a mean in-plane resolution of 0.7 mm (range: 0.3–1.0 mm) and a through-plane resolution of 6.2 mm (range: 3.0–30.0 mm). Total brain, ventricle, and WMH segmentations were accomplished using deep learning methods described in detail previously (Schirmer et al., 2019; Dubost et al., 2020). Briefly, total brain segmentation was done using a tailored 2D-convolutional neural network for clinical T2-FLAIR data. T2-FLAIR image intensities were normalized and scaled. Successively, WMH and ventricles were automatically segmented using distinct convolutional neural network frameworks. A total of 1,353 patients were excluded after final quality control of all T2-FLAIR images and respective segmentations; this control process is described in great detail in a previous publication (Schirmer et al., 2019). To capture the underlying processes of SVD in brain parenchyma not overtly affected by WMH, we computed masks for normal-appearing brain parenchyma by subtracting ventricles and WMH masks from total brain masks, resulting in 5,031 masks. To remove any stray voxels that could impact radiomics extraction, a morphological opening operation with a 3 × 3 voxel kernel was performed on the final masks. Among those 5,031 patients, 868 were excluded for missing major clinical data (age and sex), remaining missing values (89 patients included, see Supplementary Table 2) were imputed using medians of distributions. As a result, a total of 4,163 patients were included across 17 different sites.

### Radiomic Feature Extraction

Radiomic features were extracted using the open-source toolbox PyRadiomics V2.2.0. The full list of the radiomics extraction parameters can be found online at https://github.com/MBretzner/WMH_radiomicSign.

Briefly, to account for the discrepancy in voxel sizes and to reduce unwanted variance that could be originating from differences between centers and scanners, all features were extracted in-plane from down-sampled 1 mm × 1 mm × 6 mm T2-FLAIR images. Quantization was set to a fixed bin width of 5. Features extraction was performed outside of WMH on native and pre-filtered images. Filters included Laplacian of Gaussian (LoG) filters (with sigmas of 1, 2, and 3 mm), wavelet decompositions, and 2D Local Binary Patterns (2D-LBD). For each patient, 118 features were computed including mask statistics, shape features, first-order histogram statistics, GLCM (Gray Level Co-occurrence Matrix) features, GLRLM (Gray Level Run Length Matrix), GLDM (Gray Level Dependence Matrix), and NGTDM (Neighboring Gray Tone Difference Matrix) features. Exhaustive and didactic descriptions and formulas of every radiomic feature and filter can be found online at https://pyradiomics.readthedocs.io/en/latest/features.html. As a result, we extracted 763 rotation invariant radiomic features per patient.

### Machine Learning Approach to Build the Radiomic Signature of the WMH

To account for cerebral size differences, each WMH volume was divided by the corresponding brain volume to obtain a percentage of WMH per total brain volume. As the resultant distribution was highly skewed, it was transformed using a Box-Cox transform and is referred to as “WMH burden” in the next paragraphs.

To address the high dimensionality of the data, prediction of the WMH burden was done using an ElasticNet linear regressor. Since ElasticNet coefficient estimates are not scale-invariant, we standardized predictors, i.e., radiomics variables, to be 0 centered and have variances of the same order.

Radiomics-based predictions of WMH burden were performed in 30-times repeated nested fivefold stratified cross-validation scheme, resulting in a total of 24,990 out-of-sample predictions. Predictions were plotted against ground truth values, and *R*^2^ values were computed with standard deviation.

To better understand the role of each class of radiomics and to rule out an association based solely on the size of the extraction mask and thus reflecting only atrophy, an ancillary prediction of the WMH burden was performed using only the radiomics features that only reflected the size and the shape of the analyzed brains. As NIHSS has been shown to be a surrogate marker of stroke volume, and to assess a hypothetical impact of stroke lesions on radiomics features, residual of the WMH burden predictions were studied per NIHSS score when available (Tong et al., 1998). To explore the potential impact of data heterogeneity across all sites, a second ancillary analysis was performed predicting WMH burden in a leave-one-site-out cross-validation scheme.

The shrinkage ability of the ElasticNet regressor was leveraged to select the most predictive features of the WMH burden. The radiomic signature of the WMH was built with the features that were consistently selected across each of the 30 repetitions and therefore represented the most robust and stable predictors of WMH burden.

### Understanding the Textural Footprint of Clinical Phenotypes

Association of clinical variables and the radiomic signature of WMH burden were done *via* CCA, which allows studying two multivariate variable sets concomitantly (Hotelling, 1936; Wang et al., 2020). Indeed, traditional analyses explore relationships between many to one variable, whereas CCA can study complex many-to-many correlations, truly leveraging the power of multivariate datasets. CCA can be conceived as similar to principal component analyses in the way that each side of the data (here clinical and radiomics) undergoes a factorization into a latent representation of the variables, called canonical variates (CV). The canonical correlation score of a canonical function represents the correlation between the two CV that composes it. To extract each canonical function, CCA finds combinations of factors of the two sets so that they are maximally correlated. Canonical loadings represent the correlations between variables and their latent representation (CV) and can be interpreted as the relative contribution of variables to the variates: a variable with a large loading has more impact on a variate than a variable with a smaller loading.

Radiomic features and continuous clinical variable (age) normality was assessed using the Shapiro–Wilks test and, if needed, were transformed using the R toolbox *BestNormalize* (Peterson and Cavanaugh, 2020). Significance of canonical correlations was determined *via* permutation testing (1,000 permutations) and assessed using Wilks’ Lambda computed with Rao’s F-approximation, *p*-values were corrected for multiple testing with Benjamini–Hochberg procedure (Hotelling, 1936; Wang et al., 2020). Explained variances of the canonical functions were calculated and figured in a scree plot. Loadings were calculated to discover and characterize the impact of clinical and radiomic features on each canonical function and thus to provide support for the interpretation of the relationship between the radiomics and clinical domains.

Overall, the goal of CCA is to find underlying representations that best describe the correlations between the two multi-dimensional datasets. Thus, this technique permits the estimation of the sources of maximal covariance between the clinical and the radiomics domains, highlighting the subvisible contribution of cardiovascular risk factors to T2 FLAIR imaging.

### Code Availability

Radiomic features extraction, feature selection, and machine learning analyses were performed in python 3.7.6 using the toolbox *scikit-learn* (Pedregosa et al., 2011). CCA was performed in R V1.3.1056 using the toolboxes *CCA*, *vegan* (González et al., 2008; Oksanen et al., 2019). The complete codes used to perform the radiomics extraction as well as the extraction parameters and the data analysis are available here: https://github.com/MBretzner/WMH_radiomicSign.

## Results

### Population

All patients included in MRI-GENIE have suffered an ischemic stroke. Population demographics are shown in Table 1. The mean age was 62.8, and there were 42% females, median WMH volume was 4.2 mL [interquartile range (IQR): 1.4–11.2]. Admission NIH stroke scale (NIHSS) was available for 2,234 (53.7%) patients; median NIHSS was 3 (IQR: 1–6). Comparison of included and excluded patients’ available clinical characteristics is shown in Supplementary Table 2. Briefly, excluded patients were younger, had less hypertension, less coronary artery disease (CAD), less AF, but had more prior strokes. There was no difference in sex or diabetes status. Importantly NIHSS scores did not differ.

**TABLE 1:** Demographic and clinical characteristics of the study population (*n* = 4,163).

Age	Mean (SD)	62.8 (15.0)
Female	n (%)	1,748 (42.0%)
Hypertension	n (%)	2,825 (67.9%)
Diabetes mellitus	n (%)	687 (16.5%)
Atrial fibrillation	n (%)	595 (14.3%)
Coronary artery disease	n (%)	772 (18.5%)
History of smoking	n (%)	1,331 (32.0%)
Prior stroke	n (%)	539 (12.9%)
WMH volume	Median (IQR)	4.2 mL (1.4–11.2)
NIHSS*	Median (IQR)	3 (1–6)

NIHSS, NIH Stroke Scale. *NIHSS was available for 2,234 (53.7%) patients included. “Prior stroke” refers to a stroke preceding the one that led to the inclusion in the study.

### Building the Latent Radiomic Signature of the WMH Burden

**FIGURE 1:**
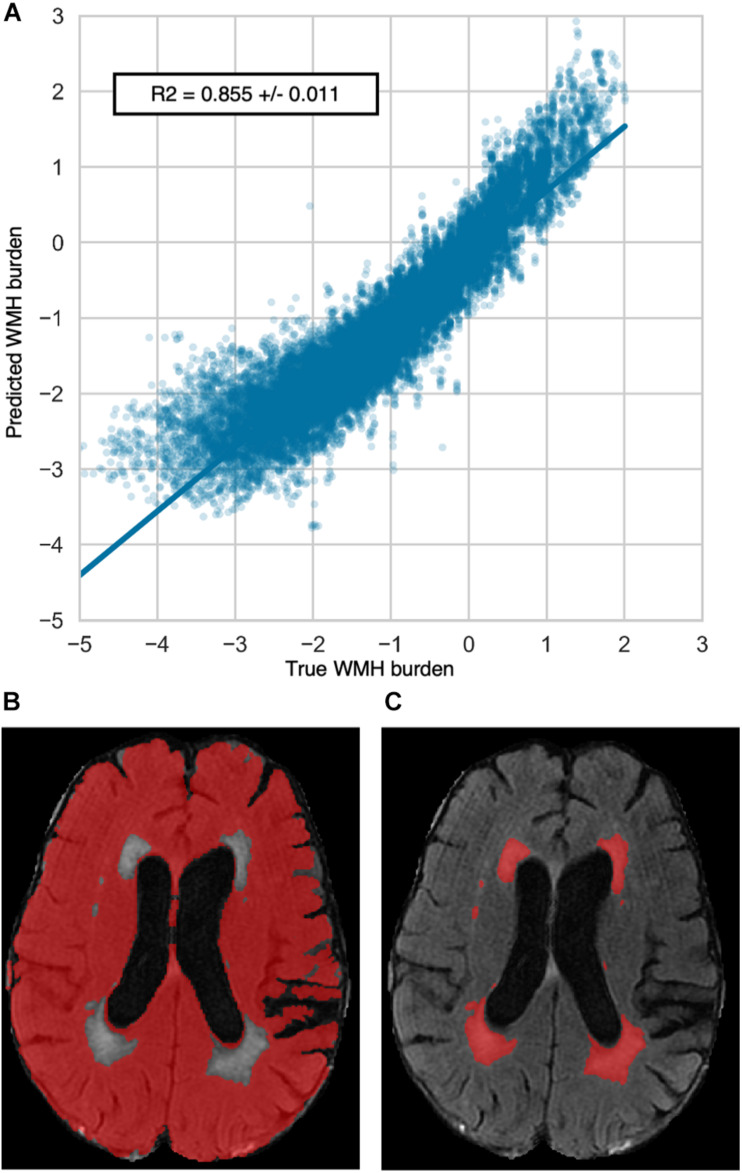
Repeated out-of-sample cross-validated predictions of WMH burden. **(A)** Predictions of the WMH burden resulted in a coefficient of determination of *R*^2^ = 0.855 ± 0.011. Predicted and true WMH burdens show negative values due to the Box-Cox transformation of the WMH burden distribution. The bottom panels provide an illustrative example of a radiomics extraction mask **(B)** and a WMH mask **(C)**.

The coefficient of determination of the repeated out-of-sample cross-validated predictions of the WMH burden was *R*^2^ = 0.855 ± 0.011 (Figure 1). The average (SD) number of selected features per repetition was 150.3 (5.6). These features represented the most relevant ones in the prediction of WMH burden. To reduce the redundancy and multicollinearity of radiomic features, we built a signature of the WMH burden by only including the features that were systematically selected in every repetition. This step resulted in the automatic selection of 68 features, which are referred to as the “radiomic signature of WMH.” These features are listed in Supplementary Table 1.

Prediction performance of the WMH burden using radiomics that only capture the shape and size of the extraction mask but not voxel intensities was substantially lower with an *R*^2^ of 0.41 ± 0.03. The analysis of the residual of the WMH burden predictions per available NIHSS score showed no trend suggesting an impact of stroke lesions on the predictions (Supplementary Figure 1).

Results of the predictions recursively holding one site out are reported in Supplementary Table 3. Distributions of patients Age and WMH volume per site are reported in Supplementary Figure 2. Briefly, when the center held-out was showing large clinical differences (mainly younger patient and/or neglectable loads of WMH), prediction performances were lower.

### Clinical Phenotypes Captured by Radiomics

Aiming to discover possible links between clinical phenotypes and textural features of the radiomic signature of WMH burden, we performed a CCA.

**FIGURE 2:**
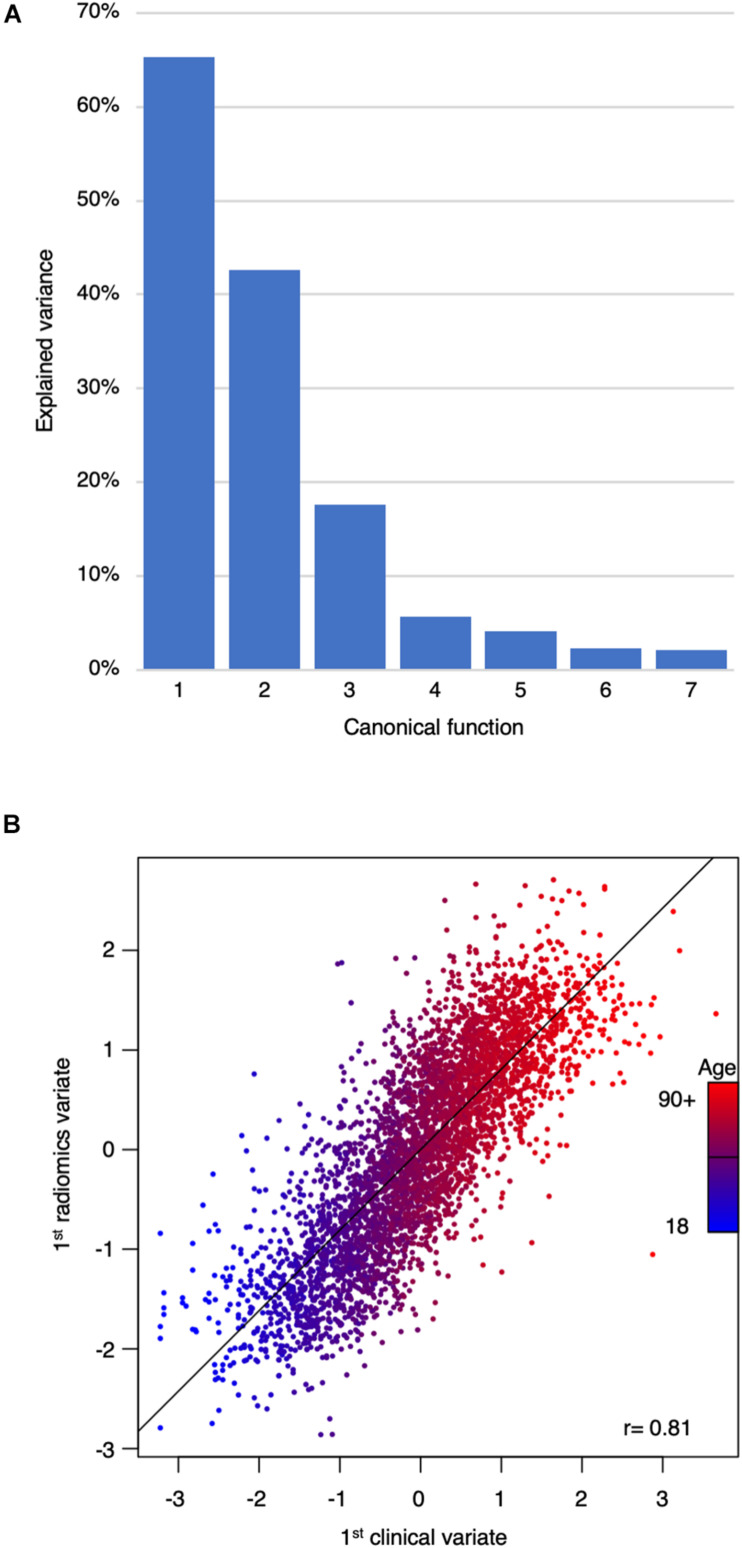
Scree plot of the explained variance per canonical function and correlation plot between the first clinical and radiomic variates. **(A)** Scree plot of the explained variance by canonical functions. **(B)** Correlation plot of the first clinical and radiomics canonical variates. Each dot represents a patient and is colored according to age. The first canonical function mainly represented age. There was a very strong correlation between the clinical and the radiomics variates of *r* = 0.81.

**FIGURE 3:**
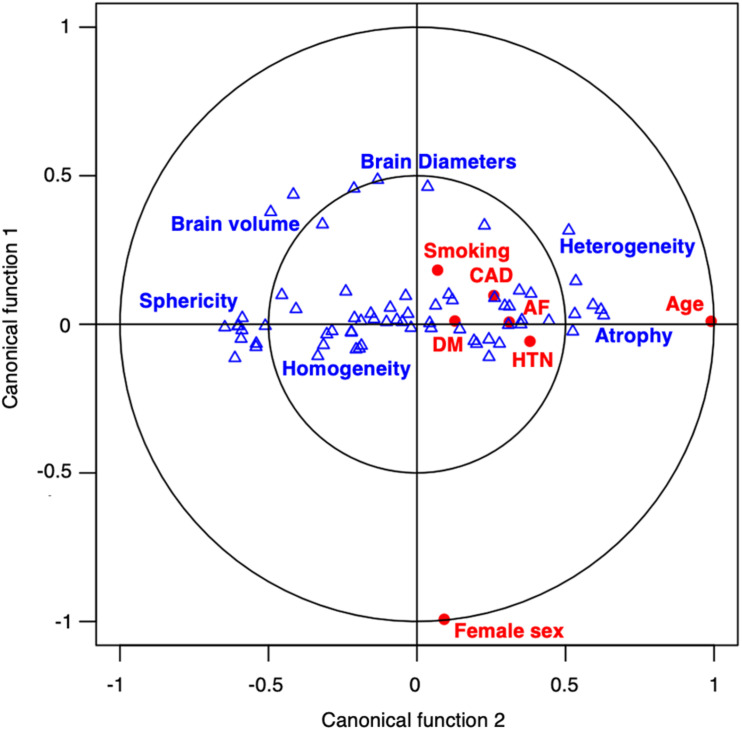
Bi-loading plot of the variables projected over the two first canonical functions. A bi-loading plot graphically represents both the correlation of variables with canonical functions, and the correlation between variables of each set. The position of a variable relative to an axis describes the strength of the correlation of that variable with the axis, the closer to the outer circle, the stronger the correlation. Clinical variables (red dot) and radiomic features (blue triangle) are positively correlated if close or negatively correlated if diagonally opposed. Blue tags were positioned next to correlated radiomic features representing common textural concepts. On T2 FLAIR images, younger patients had larger brains and more homogeneous brain tissue (left side of the plot) whereas older patients had more atrophic and heterogeneous brains (right side of the plot).

The CCA could identify seven canonical functions (CF 1–7) correlating the radiomics with clinical variates. All seven canonical functions were significant (False discovery rate corrected *p*-values CF_1__–__6_ < 10^–3^; CF_7_ = 0.012) with respective canonical correlations of 0.81, 0.65, 0.42, 0.24, 0.20, 0.15, and 0.15. Figure 2 contains the scree plot of the explained variance of each CF and the correlation plot of the clinical and radiomic variates of the first canonical function with patients points colored according to their age. Loadings of the clinical and the five most impactful radiomic variables (highest loadings) of the first two canonical functions are reported in Table 2. The bi-loading plot in Figure 3 provides a graphical interpretation relationship between the most impactful variables of the first two canonical functions. Loadings of the clinical variate of all canonical functions are shown in Table 3; loadings of the radiomics variate are presented in Supplementary Table 1. Variables that share the same direction along a given function have a positive covariance, whereas variables that show opposing directions have negative covariance. The magnitude of the loading reflects the strength of the association.

**TABLE 2:** Clinical and most impactful radiomic loadings of the first two canonical functions.

Clinical loadings			Radiomics loadings		

	CF 1	CF 2		CF 1	CF 2
AF	0.310	0.005	LoG-1mm histogram 10 percentile	−0.254	−0.128
Age	0.990	0.008	LoG-1mm GLSZM large area high gray level emphasis	−0.747	−0.008
CAD	0.260	0.097	LoG-1mm GLSZM large area low gray level emphasis	−0.743	−0.005
DM	0.127	0.009	LoG-2mm GLDM gray level non uniformity	−0.514	0.671
Hypertension	0.381	−0.057	LoG-2mm GLRLM run variance	−0.241	−0.124
Female sex	0.089	−0.993	LoG-2mm GLRLM short run low gray level emphasis	0.734	0.097
Smoking	0.069	0.180	LoG-3mm GLRLM gray level non uniformity normalized	0.300	−0.167
			LoG-3mm GLRLM short run low gray level emphasis	0.767	0.073
			Original histogram 10 percentile	−0.733	−0.071
			Original GLRLM run length non uniformity	0.662	0.221
			Original GLRLM run length non uniformity normalized	0.658	0.051
			Original GLRLM run variance	−0.801	−0.013
			Original shape major axis length	−0.263	0.696
			Original shape maximum 2D diameter column	−0.162	0.745
			Original shape mesh volume	−0.608	0.581
			Original shape minor axis length	0.046	0.709
			Original shape sphericity	−0.759	−0.172
			Original shape surface volume ratio	0.778	0.044
			Wavelet-LH GLSZM Small area high gray level emphasis	−0.413	−0.161

*AF, atrial fibrillation; CAD, coronary artery disease; DM, diabetes mellitus; LoG, Laplacian of Gaussian; GLCM, Gray Level Co-occurrence matrix; GLRLM, Gray Level Run Length Matrix; GLDM, Gray Level Dependence Matrix; NGTDM, Neighboring Gray Tone Difference Matrix. Loadings assess the relative contribution of a variable to a canonical function.*

**TABLE 3:** Clinical loadings for all seven canonical functions.

	Clinical loadings						

Canonical function	1	2	3	4	5	6	7
AF	0.310	0.005	0.102	0.020	0.313	−0.596	0.663
Age	0.990	0.008	0.096	0.080	−0.045	0.034	0.017
CAD	0.260	0.097	0.169	−0.157	−0.214	−0.744	−0.521
DM	0.127	0.009	−0.443	−0.117	0.781	−0.050	−0.401
Hypertension	0.381	−0.057	0.067	−0.916	0.055	0.058	0.030
Female sex	0.089	−0.993	−0.015	0.056	−0.004	−0.030	0.039
Smoking	0.069	0.180	−0.903	−0.055	−0.347	−0.132	0.081

*AF, atrial fibrillation; CAD, coronary artery disease; DM, diabetes mellitus. A high loading coefficient implies a higher contribution to a canonical function. For instance, age was the most important variable when establishing the clinical variate of the first canonical function.*

## Discussion

Radiomic features, extracted outside of the visible WMH, captured latent characteristics of WMH and could accurately predict WMH burden. Upon further analysis, seven distinct combinations of radiomics features were associated seven distinct combinations of clinical traits relevant to WMH, such as age, sex, hypertension, history of smoking, DM, and CAD. Therefore, the methods presented here provide new tools to help to understand and quantify the microstructural portion of the parenchymal deterioration due to SVD in stroke and give a radiological snapshot of brain health. Importantly, our analyses relied on basic T2-FLAIR images, as commonly acquired in clinical routine and thus do not require any advanced, more costly additional imaging sequences.

White matter hyperintensities represent a cardinal feature among radiological manifestations of brain aging and SVD. However, DTI-(Etherton et al., 2016) and PWI-based studies suggested (Promjunyakul et al., 2018) that WMH represent an end-stage macrostructural injury, embodying a surreptitious disease altering brain parenchyma. Our results support the hypothesis of WMH penumbra in cerebral SVD with a continuum between visible and invisible parenchymal damage (Maillard et al., 2011; Wardlaw et al., 2013). A major caveat of traditional advanced imaging biomarkers is their acquisition. Indeed, DTI sequences are rarely acquired routinely because of long scanning times, and PWI necessitates the injection of Gadolinium-based contrast agents. In contrast, our method can capture parenchymal microstructural integrity and hence, promises to replace additional dedicated imaging as a candidate approach to follow-up SVD progression in the clinic.

Cerebral atrophy has been shown to be associated with WMH burden (Aribisala et al., 2013). However, little is known about the relationship between the texture of the brain and WMH accumulation. By showing a twofold improvement of predictions leveraging exhaustive textural information compared to predictions restricted to radiomics describing only shape and size, our findings suggest that, beyond atrophy, textural analysis of the brain might better document WMH related damage of the brain. Therefore, one hypothesis would be that radiomics might be able to capture early-stage infra-radiological abnormalities prior to their evolution into irreversible cerebral loss, potentially bearing implications for future studies targeting WMH progression prevention.

By means of our CCA, we estimated the associations between the radiomic signature of WMH and SVD risk factors. The influence of cardiovascular risk factors on brain tissue was previously investigated in neuropathology and advanced imaging studies yet was rarely described by analyzing the texture of conventional imaging (Gouw et al., 2011; Wardlaw et al., 2013). Our work complement and support previous studies on MRI textural analysis applied to SVD by Valdés et al. (2017) on gadolinium-enhanced T2-FLAIR, Bernal et al. (2020) on dynamic spectral gadolinium-enhanced T1 weighted imaging, Tozer et al. (2018) on T1 and T2-FLAIR cognitive textural biomarkers, and Shu et al. (2020) and Shao et al. (2018) who could predict the progression of WMH using radiomics extracted from, respectively, T1-FLAIR and T2-FLAIR images. Our analyses were based on a large collection of clinical T2-FLAIR images, a routine MRI sequences acquired during both acute screening and follow-up of patients with stroke and cerebrovascular disease. Therefore, it argues for the overall clinical relevance of radiomics in stroke and SVD.

Age was the clinical aspect correlating most strongly with the radiomic signature of WMH burden and is a well-established predictor of WMH (Rost et al., 2010; Giese et al., 2020). Similarly, blood-brain barrier studies using PWI highlight an age-associated increased leakage of contrast agents within WMH, but also beyond, in NAWM, showcasing a possible preclinical pathogenic step leading to cognitive decline (Wardlaw et al., 2017). Our findings also suggest the presence of age-related subvisible abnormalities that can notably be quantified on structural T2-FLAIR images. Radiomic features describing atrophy (brain size and lower sphericity) and T2-FLAIR heterogeneity, were the most strongly correlated with age. On the first canonical function, age was the main variable, however, HTN, AF, and CAD were also moderately represented, painting the picture of vascular pathological brain aging. The heterogeneity and hyperintensities of the parenchyma could have maybe captured lacunes, enlarged perivascular spaces, or microbleed, which is, along with WMH, radiological hallmarks of SVD (Wardlaw et al., 2013). Radiomics presented here could therefore portray a representation of a pathological brain aging process in stroke patients, depicting atrophic and heterogeneous parenchyma.

The second canonical function illustrated sex differences in tissue aspects in T2-FLAIR. The association of the radiomic signature with sex was mainly driven by shape radiomics capturing differences in brain size. This finding remains, however, independent from age-related atrophy since canonical functions analyze the unexplained variance from the previous function. Nevertheless, the female sex was also associated with greater linear edge density (GLRLM after LoG filtering), which might indicate some sex-specific textural differences in the loss of microstructural integrity, as suggested in DTI with previous findings reporting sex-specific fractional anisotropy values (Etherton et al., 2017b).

The third canonical function captured a profile representing mainly patients with a history of smoking, and, to a lesser extent, diabetes, which shared common textural features describing more high spatial frequency changes in intensities which could represent diffuse and fine heterogeneity throughout the brain.

The fourth canonical function characterized a specific relation between hypertension and some textural features highlighting inhomogeneity on a lower spatial frequency after wavelet decomposition, thus describing a patchy texture. Since no other cardiovascular risk factor was represented on this dimension, it describes an age-independent specific textural manifestation of hypertension on T2-FLAIR.

Diabetes mellitus was mainly represented in the fifth dimension, correlating with textural features that illustrated overall less hyperintense parenchyma and especially those obtained after filtering with LoG filters. Since those filters are known to act as blob detectors, they potentially captured isolated islets of damage.

The sixth canonical function related the presence of CAD and AF to a more homogeneous texture, which was, however, combined with a high impulse response to the LoG filters of 1, 2, and 3 mm sigma that could signify the presence of spots of subvisible damage of varying size of presupposed embolic origin. On the contrary, the seventh canonical function pictured the differences separating AF from CAD and DM patients, where AF patients seemed to exhibit more patches of high spatial frequency intensity changes, which could represent zones of subtly lesioned brain.

Diabetes and AF were represented by several dimensions meaning that the diseases in question could manifest in several distinctive aspects or stages in our data. Conditional factors that could influence such diversity in presentations include the relative control of disease by treatment or lifestyle, the patient’s stage of disease severity, genetic predispositions, and endophenotypes of varying severity.

As with any work on radiomics, the main pitfall remains the *curse of dimensionality*, which refers to a very high number of variables. Consequently, one of the strengths of our study was the available sample size, allowing us to truly leverage both machine learning methodologies and multivariate modeling to select and characterize relevant radiomic variables in a data-driven fashion. In fact, to date and to the best of our knowledge, this is the largest radiomics study performed on any topic. Previous work on radiomics of SVD studied smaller datasets (<250 participants) and thus did not permit powerful unsupervised feature selections (Valdés et al., 2017; Tozer et al., 2018; Bernal et al., 2020). Another added value of the present study is its multicentric design. Our study is the first to explore radiomics of SVD in a large and multicentric population including diverse ischemic stroke cohorts with patients presenting a large spectrum of age and WMH burdens. While the heterogeneity of our dataset could be perceived as a limitation for prediction performances, we think it is on the contrary strength of our study. Indeed, our algorithm was trained on very diverse patients therefore theoretically increasing its chance of success if applied to an external dataset. By implementing multiple measures, such as down-sampling and intensity normalization, to prevent differences originating from acquisition parameters discrepancies, we could reach homogeneous results across all centers while capturing relevant sources of variance, as depicted by the low error of our WMH burden predictions. Another source of unwanted variance in radiomics analyses is segmentation. Indeed, underestimation of the WMH burden, brain parenchyma, or ventricles could have impacted our radiomics based WMH burden prediction. However, we here built upon previous results obtained with state-of-the-art deep learning-based, fully automated segmentation methods that could produce consistent outlines of brains, WMH, and ventricles from T2-FLAIR (Schirmer et al., 2019; Dubost et al., 2020). Preventive measures we implemented, especially down-sampling and intensity normalization, may have come at the cost of losing pertinent information. However, that impact might have been mitigated thanks to our large sample size. We thus emphasize the capital importance of international collaborations, such as the MRI-GENIE consortium, to gather large datasets, especially in the era of quantitative imaging and personalized medicine.

### Limitations and Future Directions

We acknowledge several limitations; first, stroke lesion outlines were not available and thus not accounted for. Overall, the median size of ischemic stroke lesions in this cohort is expected to be small, as the median NIHSS was 3. Moreover, the radiomic analysis conducted here provides a single value per radiomic variable per patient, averaging the textural presentation over the whole extraction zone and thus largely decreasing the impact of small lesions. Regarding large lesions, the corresponding perturbated radiomic value could have been assimilated to an outlier and then mitigated by the ElasticNet model, which includes an L1 regularization that improves its robustness to extreme values. The absence of clear trend in the analysis of the residual of WMH burden prediction per NIHSS score is in favor of a limited impact of stroke lesions on the predictions. Other SVD imaging features were also not accounted for, such as microbleeds or enlarged perivascular space, which have been previously linked to radiomic features (Valdés et al., 2017; Tozer et al., 2018).

Secondly, we could not study the relationship between radiomics and other known WMH biomarkers such as dyslipidemia.

Thirdly, we here suggest a novel biomarker to assess the structural integrity of the brain on routine T2-FLAIR imaging. However, as with every new biomarker, the results presented here would need external validation, especially to appreciate the robustness of the features included in the radiomic signature of WMH.

Fourthly, a substantial number of patients were excluded from the analysis because of failed segmentation mainly due to image quality. While this might bias the analysis, it also highlights the challenges of processing clinical imaging in a real-world setting.

Lastly, radiomics were extracted outside of the WMH but not specifically within the white matter. Future research could evaluate the impact of co-registration and resampling on radiomics of SVD, then benchmark radiomics of NAWM against more traditional DTI metrics in the prediction of clinical outcomes and therefore provide a more straightforward method to quantify microstructural integrity.

## Conclusion

In a large cohort of ischemic stroke patients, we demonstrated that radiomic features predicted WMH burden and were associated with clinical factors. By applying machine learning methods to radiomics analyses of T2-FLAIR images from a large multi-site ischemic stroke cohort, we could characterize the latent expression of SVD that extends beyond the visible WMH and subsequently uncover links associating cardiovascular risk factors to distinct textural patterns. Radiomics analysis may hold promise to become a cost-effective tool to quantify microstructural damage on routinely acquired images in the follow-up of SVD and stroke patients, once externally validated.

## Data Availability Statement

Data will be made available upon reasonable request and with approval from local IRBs, to replicate the results presented in this manuscript. Requests to access the data should be directed to NR: nrost@partners.org

## Ethics Statement

The studies involving human participants were reviewed and approved by the MRI-GENIE project and by the MGH Institutional Review Board (IRB, Protocol #: 2001P001186 and Protocol #: 2003P000836), as well as ethics boards of the collaborating institutions: (Albrecht-Kossel Institute for Neuroregeneration (AKOS) University of Rostock, Germany; University of Cincinnati (UC) College of Medicine, USA; Geisinger Institute, USA; Helsinki University Central Hospital, Finland; IMIM - Hospital del Mar, Spain; Imperial college of London, UK; Mayo Clinic Florida, USA; Medical University Graz, Austria; Sahlgrenska Academy at University of Gothenburg, Sweden; Skane University Hospital, Sweden; Leuven University Hospitals, Belgium; University of British Columbia, Canada; University of Maryland School of Medicine, USA; University of Miami, USA; University of Newcastle, Australia). All participants or health care proxy provided signed informed consent. The patients/participants provided their written informed consent to participate in this study.

## Author Contributions

MB, AB, MS, SH, ME, PR, RM, CW, RR, XL, RL, GK, and NR: conception and design of the study. MB, AB, MS, SH, ADa, KD, A-KG, ME, PR, MN, OB, JC, ADo, CG, LHo, LHe, KJ, JJ-C, SK, RL, CL, PM, CM, JMe, C-LP, AR, SR, JRs, JRq, TR, RSa, RSc, PS, ASl, ASo, TS, DS, TT, VT, AV, JC, DW, OW, RZ, BW, JM, AL, CJ, PG, and NR: acquisition and analysis of data. MB, AB, MS, ME, RM, CW, RR, XL, ReL, CG, JMe, JW, GK, and NR: drafting a significant portion of the manuscript or figures. All authors contributed to the article and approved the submitted version.

## Conflict of Interest

AR was employed by the company Centogene GmbH. The remaining authors declare that the research was conducted in the absence of any commercial or financial relationships that could be construed as a potential conflict of interest.
